# Phytochemicals in Malignant Pleural Mesothelioma Treatment—Review on the Current Trends of Therapies

**DOI:** 10.3390/ijms22158279

**Published:** 2021-07-31

**Authors:** Malgorzata Chmielewska-Kassassir, Lucyna A. Wozniak

**Affiliations:** Department of Structural Biology, Medical University of Lodz, Zeligowskiego 7/9 Str., 90-752 Lodz, Poland; malgorzata.chmielewska-kassassir@umed.lodz.pl

**Keywords:** MPM, chemotherapy, natural compounds, phytochemicals, polyphenols

## Abstract

Malignant pleural mesothelioma (MPM) is a rare but highly aggressive tumor of pleura arising in response to asbestos fibers exposure. MPM is frequently diagnosed in the advanced stage of the disease and causes poor prognostic outcomes. From the clinical perspective, MPM is resistant to conventional treatment, thus challenging the therapeutic options. There is still demand for improvement and sensitization of MPM cells to therapy in light of intensive clinical studies on chemotherapeutic drugs, including immuno-modulatory and targeted therapies. One way is looking for natural sources, whole plants, and extracts whose ingredients, especially polyphenols, have potential anticancer properties. This comprehensive review summarizes the current studies on natural compounds and plant extracts in developing new treatment strategies for MPM.

## 1. Introduction

Malignant pleural mesothelioma (MPM) is a relatively rare tumor of pleura arising in approximately 80% of cases due to prolonged occupational (rarely environmental or domestic) exposure to asbestos fibers in the past. Each year, the incidence rate of MPM increases significantly, and according to the European Society for Medical Oncology (ESMO), only within the next 20 years, the newly reported incidents of patients with MPM will be doubled, with a fivefold higher incidence in case of men [1,2]. In many European countries, the incidences of mesothelioma result from a prolonged latency period between asbestos exposure (starting in the 1970s) and cancer diagnosis [3]. Worryingly, this particular cancer type is highly aggressive and insensitive to conventional treatment, including radiotherapy, resection (in justified cases), and primary chemotherapy. Chemotherapy with antifolic analog is the first-line standard therapy—pemetrexed and cytostatic platinum, with the median overall survival reaching approximately one and a half. New targeted strategies and immunotherapies, especially immune checkpoint inhibitors, are under intensive investigation for patients who are not candidates for cisplatin-based therapy.

Additionally, due to the delayed (as a result of latent character of the disease lasting even 30 years) and very often ambiguous diagnosis (misdiagnosed with small-cell lung cancer; SCLC), the course of the illness and, in consequence, prognostic outcomes for MPM-bearing patients are significantly reduced. In the face of the dark nature of MPM, apart from improving the accurate and early diagnosis, it seems reasonable to search for novel therapeutic strategies, including natural compounds that may help manage MPM tumors. This review sheds light on the current state of research on phytochemicals investigated in MPM therapy.

## 2. Novel Targeted Therapies against Malignant Pleural Mesothelioma Cells

**Figure 1:**
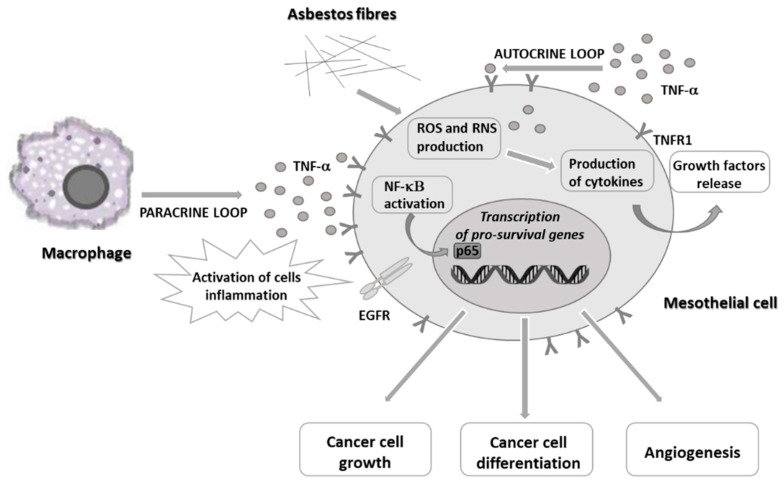
Pathogenesis of human mesothelial cells upon asbestos fibers contact. (TNF-α, tumor necrosis factor-alpha (grey dots); TNFR, tumor necrosis factor-alpha receptor (Y symbol); NF-κB, nuclear factor kappa B; ROS, reactive oxygen species; RNS, reactive nitrogen species; EGFR, epidermal growth factor receptor).

The prolonged inhalation of asbestos fibers leads to chronic inflammation within the pleura and, as a consequence, facilitates carcinogenic processes. Asbestos fibers mechanically damage the integrity of mesothelial tissue, leading to a local inflammatory response with observed overproduction of free radicals (reactive oxygen species, ROS, and reactive nitrogen species, RNS) and pro-inflammatory cytokines [4,5]. At the beginning of the inflammatory process, the tumor necrosis factor-alpha (TNF-α) and its downstream nuclear factor kappa B (NF-κB) signaling pathway play a critical role. Upon mechanistic injury, mesothelial cells secrete high-mobility group bax 1 protein (HMGBP1), which stimulates macrophages recruitment in the surrounding space of the damaged mesothelial cells and releases excessive amounts of TNF-α mediator [6]. TNF-α acts directly with its receptor TNF-R1 expressed on the surface of human mesothelial (HM) cells [7]. In turn, this interaction stimulates HM cells into further autocrine release of TNF-α, which finally activates and accelerates NF-κB downstream signaling, mainly pro-survival gene transcription by the p65 subunit of NF-κB, skipping an apoptosis process of the injured cells [8]. Consequently, HM cells intensively divide rather than dying, causing mistakes in the uncontrolled division that accumulates DNA synthesis, leading to malignant mesothelial cell transformation (Figure 1).

**Figure 2:**
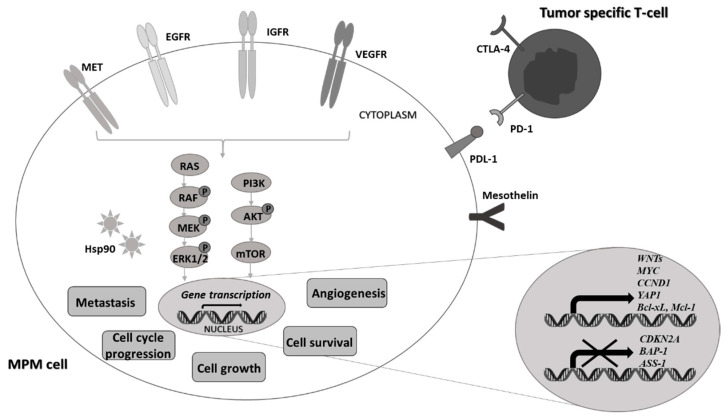
Molecular pathways involved in the pathogenesis of MPM—potential targets for therapy. (EGFR, epidermal growth factor receptor; IGFR, insulin-like growth factor receptor; VEGFR, vascular endothelial growth factor receptor; MET, hepatocyte growth factor receptor; PDL-1, programmed cell death protein ligand-1; CTLA-4, cytotoxic T lymphocyte-associated protein; PD-1, programmed cell death protein-1; Hsp90, heat shock protein-90; P, phosphorylated form of signaling molecules).

Several alterations in genes and molecular pathways related to mesothelial cell proliferation, susceptibility to apoptosis, and angiogenic processes are observed under MPM carcinogenesis [9]. The most frequent in MPM pathogenesis are genetic mutations in tumor suppressor genes (including neurofibromin 2 (NF2, merlin), cyclin-dependent kinase inhibitor 2A (CDKN2A), which shares a locus for both p16/INK4 and p14/ARF, and BRCA-associated protein-1 (BAP-1). The observed genetic alternations in these genes play a significant role in MPM development due to their direct involvement in controlling cell shape, adhesion, and cell growth [10]. Some additional alternations involve Wnt/β-catenin signaling pathways, as well as epigenetic silencing of metabolic pathway-related genes, including arginine succinate synthase (ASS1) [11]. High expression of angiogenic growth factors (e.g., vascular endothelial growth factor, VEGF) as well as their surface receptors (e.g., VEGFR, EGFR (epidermal growth factor receptor) or MET (hepatocyte growth factor receptor) and other glycoproteins (mesothelin) is a predominant determinant of intracellular signaling pathways in carcinogenic MPM cells [12]. These molecular factors and their downstream molecular pathways are currently intensively investigated and comprise the first line of interest in targeted MPM treatment strategy (Figure 2). However, until now, no significant effects have been observed in the case of anti-angiogenic drugs (in either VEGF/VEGFR or PDGF/PDGFR (platelet-derived growth factor/PDGF receptor targets)) or pro-apoptotic therapies including histones deacetylases inhibitors (HDAC) and PI3K/AKT/mTOR pathway (phosphatidylinositol-3-kinase and alpha serine/threonine-protein kinase) [13,14]. Both targeted therapies, including anti-mesothelin, anti-MET, and anti-Hsp90 (Heat shock protein-90) antibodies, and immunotherapies with predominant immune checkpoint inhibitors (anti-CTLA-4, anti-PD-1, and anti-PD-L1) are still under clinical trials due to lack of direct recommendations of such treatment to MPM patients.

Further detailed studies are required to correlate patients’ characteristics and expression status of target molecules with the implementation of a particular therapy. The observed discrepancy between clinical trials and documented unresponsiveness of mesothelioma to most targeted agents can be explained by, first of all, the non-randomized type of studies (unselected based on unique biomarker patients) as well as the analysis of various endpoints (response rate, progression-free rate vs. overall survival data). Second, the resistance of malignant pleural mesothelioma cells to apoptosis-triggering signals can be highly individual; thus, the development of selective biomarkers of response is urgently needed. Moreover, unrelated directly to apoptotic pathways, new approaches have recently been extensively studied, including metabolite deprivation (e.g., arginine starvation of malignant mesothelioma cells) or enhancement of immune system response (including administration of oncolytic viruses and therapeutic peptide or dendritic cells vaccines) [13].

Some MPM subtypes are characterized by epigenetically lost or reduced expression of argininosuccinate synthetase-1 (ASS1), known as a rate-limiting enzyme involved in the endogenous synthesis of L-arginine in the urea cycle. Recent studies have demonstrated that the growth and viability of these so-called ASS1-negative cancer cells are highly dependent on the L-arginine level [15]. This L-arginine auxotrophic phenomenon of ASS1-negative tumors, including melanoma, hepatocellular carcinoma, and bladder cancer, has led to the development of a new therapeutic strategy using the arginine deiminase enzyme (in pegylated form; ADI-PEG20), responsible for the deprivation of L-arginine presented in the environment of the tumor cells and lethality of MPM cancer [16,17]. A Phase 1 TRAP trial confirmed positive outcomes in ASS1-deficient patients under treatment with ADI-PEG20 alone or combined with cisplatin plus pemetrexed [18,19]. The arginine starvation treatment strategy resulted in encouraging results. The randomized, double-blinded Phase 2/3 clinical trial, NCT02709512, is currently being conducted to evaluate the effectiveness of ADI-PEG20 in combinatory treatment with first-line standard therapeutics (cisplatin/pemetrexed) in patients with biphasic (mixed) or sarcomatoid subtypes of MPM. The ASS1-related targeted therapy is the first of the triplet chemotherapy combination (ADIPEMCIS) studies that causes (based on Phase 1 results) effective plasma arginine deprivation in recruited MPM patients, with similar good prognostic outcomes reflected in both the median progression-free survival (PFS) value and median overall response (OS) rate, 5.6 and 10.1 months, respectively.

Today, the ongoing predominant clinical trials are focused on immunotherapy since the immune system and immune cells play a critical role in the pathogenesis of malignant pleural mesothelioma. While the development of MPM is a consequence of mechanical damage of epithelioid cells with the concomitant chronic inflammatory process, lasting a long time in the surrounding environment of pleura, several critical immune checkpoint targets are under investigation [13,14]. The most promising targets receiving research attention are cytotoxic T lymphocyte-associated protein (CTLA-4) and a programmed cell death protein-1 (PD-1) expressed on regulatory (CD4 ‘helper’) T cells, and a programmed cell death protein ligand-1 (PD-L1) highly expressed by MPM cells. The targeted strategy against these molecules aims to restore CD8 ‘killer’ T lymphocytes’ activity and proper anti-tumor immune response [20]. However, despite the positive preliminary results, neither anti-CTLA-4 nor anti-PD-L1 provides successful benefits in a single treatment strategy; thus, further validation of clinical outcomes is still required in combinatory studies of a larger randomized trial. The most hopeful seems to be the anti-PD-1 immunotherapy in unresectable MPM cases [21].

Some encouraging results have also been seen in utilizing chimeric monoclonal anti-mesothelin antibody (amatuximab) in the clinical trial. The overexpression of mesothelin, observed mainly in epithelial cancers, seems to be related to tumorigenesis and metastasis. The preclinical studies revealed that amatuximab induced antibody-dependent cellular cytotoxicity of mesothelin-expressing tumor cells in in vitro studies and significantly reduced mesothelin-positive tumors in the xenograft model. The Phase 2 clinical study demonstrated that amatuximab treatment was well-tolerated by patients with unresectable MPM, and its synergy with chemotherapeutics, cisplatin, and pemetrexed, improved the median overall survival. However, it did not influence the progression-free survival rate [22].

Moreover, in another study, Hassan et al. firmly pointed out the potential induction of innate and adaptive response, mainly CD4+- and CD8+-specific T-cells, to non-virulent *Listeria monocytogenes* bacterium-expressing mesothelin surface glycoprotein (CRS-207 construct) [23]. A Phase 2 study performed on patients with an advanced stage of mesothelioma has shown that CRS-207 was well-tolerated, with the observed reduction of tumor size. The CRS-207 therapy alone and before the standard chemotherapy rearranges the number of circulating immune cells, providing an effector response of modified tumor microenvironment to tumor cells.

**Figure 3:**
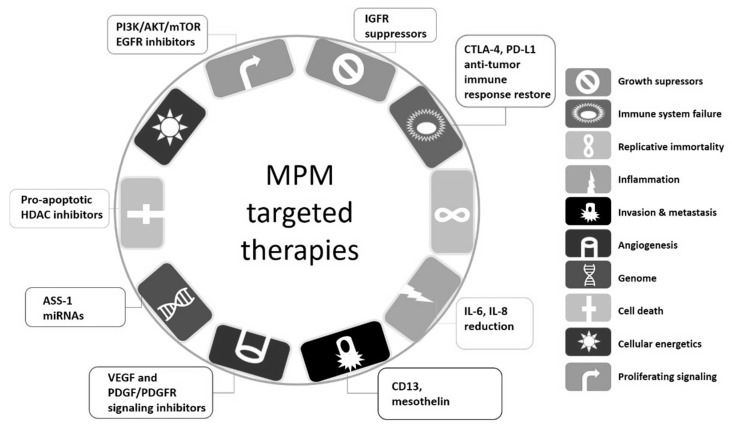
Mapping targets in the MPM therapy (based on Hanahan D, Weinberg RA. 2011 [25]).

In summary, currently, there are several widespread targets in studies of mechanism and MPM therapy, as collected in Figure 3. They focus on inflammation reduction (IL-6 and IL-8), immune system failure (by blocking CTLA-4 and PD-1 receptors on unresponsive T-cells), invasion and metastasis (by anti-mesothelin as well as anti-CD13 expressed on tumor endothelium), but also angiogenesis (via an anti-VEGF approach) as well as direct cell death (by HDAC inhibitors, which induce tumor-cell-selective expression of pro-apoptotic genes). The miRNAs [24] and ASS-1 remain obvious targets for genomic alterations, whereas changes in cellular energetics, oxidative stress, or processes of replicative immortality still remain new and are not well-investigated and incorporated into the trials. Therefore, further in-depth research on possible targets is still ongoing, in both in vitro and in vivo models.

## 3. Polyphenols and Natural Compounds in Cancer Treatment

Numerous plant-derived dietary supplements have been used for years as a natural support of the body in handling inflammatory processes, treating various metabolic diseases, or improving the immune system. The extracts obtained from natural sources, including green tea, grape seeds, and borage seeds, are characterized by a rich polyphenol composition and well-documented antioxidant properties [26].

Polyphenols are secondary metabolites that belong to the phenolic family of naturally occurring compounds present in plants’ roots, leaves, and seeds. Chemically, they consist of phenol structures divided into several subclasses depending on the number of phenolic rings and the arrangement of substituent groups (hydroxyl, alkyl, and glycosyl groups) that bind to these rings. In general, polyphenols are classified into flavonoids (consisting of flavonols, flavones, flavanols, flavanones, isoflavones, and anthocyanidins) and non-flavonoid compounds with phenolic acids, stilbenes, lignans, and other polyphenols. The main representatives of these phytochemicals are listed in Table 1.

**Figure 4:**
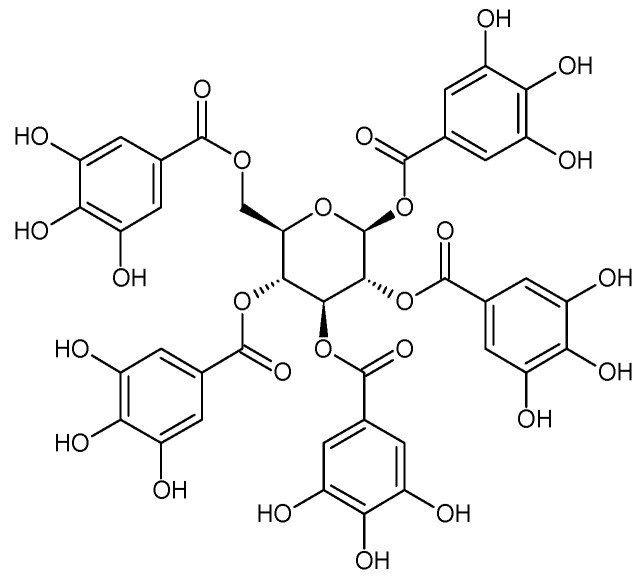
Penta-O-galloyl-β-D-glucose polyphenol (PGG).

In the light of the revealed significant antioxidant activity and protective role in the whole human body of polyphenols and other natural compounds, phytochemicals are increasingly considered dietary supplements in anticancer therapy [27]. Recent studies demonstrated that polyphenol-rich plant extracts abolish common side effects, such as cardiotoxicity and nephrotoxicity, associated with standard chemotherapy in tumor-bearing patients [28]. For instance, Zhang et al. summarized in detail the beneficial results of penta-O-galloyl-β-D-glucose (PGG), a gallotannin that is a penta-gallic acids ester of glucose (Figure 4), not only in diabetes treatment but particularly in anticancer therapies including prostate, lung, and breast cancers. PGG can reveal antitumor potential through several mechanisms, including anti-angiogenic and metastatic properties (through the VEGF inhibition and direct diminishing the activity of matrix metalloproteinase-9, MMP-9), antiproliferative actions (through S-phase perturbation and immediate G0/G1 growth phase arrest), and the anti-inflammatory and antioxidant role in normal, healthy cells (by scavenging reactive oxygen species (ROS) in a dose-dependent manner) [29]. It seems that several key carcinogenic molecular players are targets for not only PGG antitumor activity but also other phenolic derivatives. These include nuclear factor-kappaB (NF-κB) and tumor suppressor genes (P53 and retinoblastoma, Rb) [30].

Interestingly, PGG may also reverse drug resistance in treated malignant cells by directly inhibiting P-glycoprotein, the cell membrane protein responsible for rejecting therapeutic drugs out of cancer cells [31]. Furthermore, Ryu et al. demonstrated for the first time that PGG decreased the cytotoxicity effect of cisplatin in renal cells by reducing reactive oxygen species generation [32]. Additionally, the increased sensitization of Caki-2 renal cancer cells to the antitumor activity of cisplatin in the presence of PGG was observed, which strongly indicates a positive role of PGG supplementation strategy.

Recently, there have been many reports on the beneficial anticancer properties of polyphenolic compounds. The molecular mechanisms of various valuable phenolic compounds are still under deep investigation in either in vitro or in vivo models [27,29,33]. Numerous reports have shown either the modulatory effect or direct interaction of phenolic compounds with the particular oncogenes, affecting the major oncogenic signal transduction pathways involved in carcinogenesis and cancer progression. Moreover, several studies revealed the potential antitumor action of mixtures of selected polyphenolic compounds. The most commonly used combined treatment strategies with curcumin, flavonoid epigallocatechin gallate (EGCG), or resveratrol show good prognostic results in inhibiting tumor cell proliferation and arresting cancer cell growth. The promising effects of polyphenol combinations or natural extracts, in low doses, have also been seen in several cancer types, including lung cancer, breast cancer, prostate cancer, and leukemia cells [34,35,36,37]. In particular, polyphenolic compounds can be successfully administered in conjugation with standard chemotherapeutics overcoming the drug resistance phenomenon [27].

## 4. Polyphenols in Malignant Pleural Mesothelioma Therapy

Polyphenols are a diverse group of natural compounds that may act on the cells either in an antioxidative or pro-oxidative way, depending on the concentration, cell type, and operating conditions. Their biological pro-oxidative properties are essential for cancer treatment since the excessive generation of ROS leads to the breakdown of DNA, inhibiting cell growth and death. The cancer cells are more susceptible to ROS than normal ones [38]. ROS mediate apoptosis, necrosis, and autophagy processes that are desirable targets in anticancer therapies. Additionally, ROS contribute to the increased cytotoxicity of chemotherapeutic agents in combined treatment strategies with polyphenol compounds.

Curcumin, widely studied for its activity [39,40], was one of the first compounds reported to cause mesothelioma cell death, partially by direct stimulation of cell apoptosis [41]. The MPM cell growth suppression induced by curcumin took place via direct stimulation of Bax proteins and caspase-9, both engaged in initiating the apoptotic pathway. Moreover, Wang et al. revealed for the first time that curcumin was also responsible for the induction of expression of novel transducing molecules, including XIAP-associated factor-1 (XAF1) and CARP1/CCAR1 proteins, responsible for signaling to apoptosis. Finally, they demonstrated that curcumin treatment stimulated the expression of tumor suppressor protein SULF1, which could influence multiple tyrosine kinase-signaling pathways, making SULF1 a potential molecular target for MPM and other cancer treatment options. Polyphenols, including curcumin, can modulate immune cells involved in tumor development and control inflammatory processes that engage cytokines and further ROS generation [42]. Miller et al. observed that in MPM cells, curcumin enhanced activation of caspase-1, an interleukin-conversing enzyme (ICE), physiologically responsible for maturing pro-inflammatory cytokines interleukin-1β and -18. However, the authors also found a higher concentration of pro-IL-1β, which evidenced an anti-inflammatory effect of curcumin on MPM cells, leading to lytic programmed cells death [43].

Moreover, curcumin-mediated apoptosis was related to the increased Bax protein, p53 expansion, activation of pro-apoptotic caspase-9, and accumulation of cells at the Sub-G1 phase [44]. Beyond the influence on the cell cycle, curcumin treatment led to phosphorylation of ERK1/2 and p38 mitogen-activated protein kinase (MAPK), blockage of NF-κB nuclear translocation, and JNK pathway inhibition. In the Masueli et al. study, treatment of the murine model of MPM with curcumin reduced tumor development and increased the median survival of an experimental animal. Additionally, the curcumin combined with another natural compound, piperine (commercially available as C3 complex^®^ and Bioperine^®^), reduced the tumorigenic properties of mesothelioma cells, including cell proliferation, colonization, invasion, and reduced angiogenesis in a mesothelioma xenograft mouse model [45]. Under the daily intraperitoneal administration of C3 complex/Bioperine^®^, a significant delay in the growth of tumor cells was observed and a decrease in ectopic cell proliferation and the number of vessels and increased apoptotic index were revealed. Pouliguen et al. demonstrated the inhibitory effect of curcumin on mesothelioma cells’ motility and further tumor metastasis and liver colonization [46]. From the clinical point of view, the most significant advantage of curcumin is its biosafety and immediate interaction [47]. Thus, the direct intrapleural (local) administration is still further investigated as a future alternative for patients with pleural cavity tumors, including MPM [48].

Epigallocatechin-3-gallate (EGCG) is another compound extensively investigated against mesothelioma cells [49]. There are three concomitant reports that complement each other. In all cases, EGCG treatment has induced mesothelioma cell death. The studies on the mechanism of EGCG action indicated the pro-oxidative properties of this polyphenol at higher therapeutic doses. EGCG induced the formation of reactive oxygen species, including hydrogen peroxide and superoxide, outside of the cells while impairing mitochondrial membrane potential [50,51]. This flavanol decreased the MPM proliferation and led to mitochondria apoptosis only in tumor-changed cells. EGCG has also been successfully combined in reducing MPM cell viability with ascorbic acid and gemcitabine, the latter being the cytostatic antimetabolite, an analog of pyrimidine 2′-deoxycytidine [52]. The combined treatment of EGCG with gemcitabine led to non-inflammatory apoptosis of REN MPM cells by downregulation of the p65 subunit of NF-κB, restoring programmed cells death [53]. EGCG has also been shown to modulate the constitutive for tumor cells unfold protein process response (UPR) by accumulating molecular chaperone GRP78 in the endoplasmic reticulum (ER). The increased GRP78 protein in ER promotes the activation of pro-apoptotic caspases 3 and 8, together with an increased expression of transcription factors directly related to stress-responsive genes, including cyclin AMP-dependent transcription factor (ATF-4) and X-box-binding protein 1 (XBP1) [54]. These observations indicated the reasonable possibility of the therapeutic use of EGCG in MPM treatment.

The beneficial effect of resveratrol (3,5,4′-trihydroxystilbene) with clofarabine or cisplatin as chemotherapeutics was observed in the in vitro model mesothelioma MSTO-H211 cells [55,56,57]. In the presence of a low dose of the chemotherapeutic agent (clofarabine or cisplatin), resveratrol caused inhibition of MPM cell growth and initiation of apoptotic processes. Upon the ROS-mediated inflammatory process, activation of the Nrf2 transcription factor (nuclear factor E2-related factor 2) is observed. Under the physiological state, Nrf2 is ubiquitously sequestered by Keap-1 (Kelch-like ECH-associated protein). However, when MPM arises, Keap-1 undergoes a conformational change, releasing Nrf2, which translocates to the nucleus and activates the transcription of anti-inflammatory genes [58]. It has been established that the increased Nrf2 level created is favorable for the MPM immune-resistant environment. Lee YJ et al. reported that the observed restoration of MPM cell sensitivity to clofarabine in the presence of resveratrol was accompanied by downregulation of Nrf2 [55]. Resveratrol contributed to overcoming the chemoresistance of MPM cells to clofarabine via a p53-dependent apoptotic pathway with significant accumulation of tumor cells in the G1 phase and increased caspase-3/7 activity [56].

On the other hand, the synergistic activity of resveratrol and cisplatin led to increased ROS production and induced apoptosis through promoted mitochondria oxidative damage events [57]. The most significant role in oncogenesis is transcription factor Specificity Protein 1 (Sp1). That protein is abundantly expressed in different tumor cells and controls the transcription of oncogenes and tumor suppressors and genes involved in cancer proliferation, differentiation, apoptosis, genetic material damage responses, and so on [59]. Thus, this particular signaling factor seems to be a promising target for anticancer management. Lee KA et al. demonstrated that resveratrol induced growth arrest of MSTO-H211 cells at the Sub-G1 phase in vitro study and directly suppressed Sp1 protein level and expression of Sp1 regulatory genes cyclin D1, p21, p27, surviving, and myeloid cell leukemia (Mcl-1) [60]. Concomitantly, Lee YJ et al. confirmed that resveratrol combined with clofarabine reduced the nuclear accumulation of Sp1 and decreased the levels of p-Akt, Sp1, c-Met, cyclin D1, and p21 in MSTO-H211 cells upon the synergistic antiproliferative [56]. Finally, resveratrol alone reduced MSTO-H211 tumor growth in a BALB/c athymic (nu+/nu+) mice animal model via the inhibition of Sp1 expression. The authors observed a decreased tumor volume and activation of pro-apoptotic signals transduction [60]. The Mcl-1 protein seems to be a direct downstream target, responsible for the observed effect of treatment of malignant mesothelioma cells with resveratrol and clofarabine, locking the apoptotic pathway. The downregulation of Mcl-1 protein after administrating the resveratrol and clofarabine formulation restored the growth-inhibitory effect and induced the caspase-3 activation pathway [61].

Analogously, quercetin, another plant flavonoid, revealed a potential inhibitory effect on human MSTO-H211 cell viability. In the study pf Chae JI et al., quercetin increased the population of Sub-G1 cells via interaction with the Sp1 transcription factor, significantly suppressing its expression at protein and mRNA levels [62]. Quercetin caused downregulation of expression of the Sp1-related genes (cyclin D1, Mcl-1, and survivin) and activated pro-apoptotic caspase-3 signals, triggering apoptotic cell death. Quercetin, in combination with cisplatin, revealed a positive mode of action. In the in vitro study on SCP212 and SCP111 cells, quercetin reduced MPM cell proliferation and activated caspase-9 and caspase-3, leading to apoptosis [63]. Additionally, the combinatory dose of quercetin and cisplatin was more effective in MPM treatment than the individual agents. Demiroglu-Zergeroglu et al. confirmed in another study that quercetin alone and in combination with cisplatin modulated the expression of genes involved in cell-cycle progression, mainly cyclins and cyclin-related molecular pathways, with parallel upregulated expression of MAPK-related genes [64]. Quercetin and cisplatin triggered S-phase cell cycle arrest via increased cyclin-dependent kinase inhibitor genes (CDI) and forced cell death by activating p38 and JNK pathways.

Epidermal growth factor receptor (EGFR) is one of the main targets in mesothelioma treatment strategies due to its overexpression reaching 75% in MPM patients. Until now, gallic acid was one of the phenolic acids that showed EGFR-dependent pro-apoptotic and antiproliferative activity in malignant pleural mesothelioma cells [65]. GA induced transient cancer cells growth arrest in the G1 phase and mitochondrial dysfunction, leading to apoptosis. The mechanism underlying its biological activity was tightly related to p38 MAPK inactivation. After GA treatment, the reduced viability of mesothelioma cells resulted in a down-regulated cyclin D and Bcl-2 gene expression and simultaneous upregulated expression of p21, genes directly involved in cell cycle progression and apoptotic signals.

Cafestol and kahweol, two diterpenes present in the beans of coffee Arabica, are among the natural compounds also investigated for MPM treatment [66]. These bioactive compounds induced MPM cell apoptosis again via downregulation of the Sp1 protein level and downstream Sp1 gene-related regulatory genes, including cyclin D, Mcl1, and survivin. Moreover, the single use of either kahweol or cafestol chemical led to increased pro-apoptotic signals in MSTO-H211 cells via the regulation of Bcl-2-related proteins (upregulation of Bax, downregulation of Bcl-xL), and activation of Bid, caspase-3, and PARP, respectively. The combinatory dose of kahweol and cafestol has a chemopreventive effect on an animal model, where a mixture of these two compounds inhibited carcinogen-activated hepatic cytochrome P450 (CYP450) and sulfotransferase (SULT) [67].

**Figure 5:**
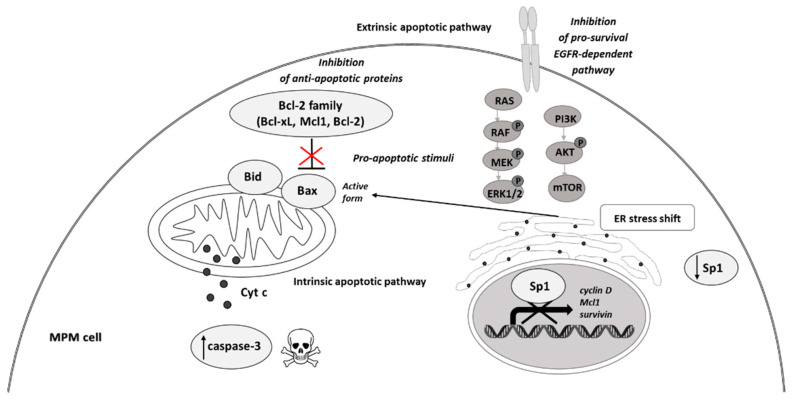
Molecular targets of natural compounds in malignant pleural mesothelioma treatment. (Cyt c, cytochrome c (black dots); P, phosphorylated form of signaling molecules).

Lee KA et al. [68] demonstrated that hesperidin, a bioflavonoid from citrus fruits, significantly reduced the Sp1 at protein and mRNA levels. Furthermore, this flavanone glycoside suppressed the viability of MSTO-H211, leading to the accumulation of cells at the Sub-G1 phase, and induced an apoptotic pathway by the cleavage of Bid, caspase-3, and PARP, as well as upregulation of Bax and reduction of Bcl-xL protein levels. Analogously, Chae JI et al. described that honokiol, an active component of the *Magnolia genus*, used in traditional Chinese medicine, similarly inhibited MPM cell growth to the above-discussed diterpenes from coffee, quercetin, and citrus-derived hesperidin via downregulation of Sp1 expression and its target transcription genes [69]. Like other above-discussed natural compounds, honokiol also led to mesothelioma cell growth arrest in the Sub-G1 phase and induced apoptosis-signaling cascade events, with observed Bax activation caspase-3 and PARP with parallel reduction of anti-apoptotic Bid and Bcl-xL proteins. The equal contribution of the Sp1 transcription factor was documented for licochalcone A (LicA), a natural compound extracted from the roots of licorice species *Glycyrrhiza* ssp. LicA is a well-known anti-inflammatory agent that protects sensitive and injured skin and serves as a beneficial component of dermo-cosmetics [70]. Kim HK et al. demonstrated that the isolated and purified LicA regulated the growth and proliferation of the investigated MPM cell lines and arrested the cell cycle at the Sub-G1 phase [71]. Furthermore, LicA down-regulated the expression of Sp1 and consequently its downstream genes, as cyclin D1, Mcl1, and survivin. Finally, as others mentioned, LicA induced the intrinsic mitochondrial apoptotic pathway by modulating pro-apoptotic and anti-apoptotic proteins of the Bcl-2 family. The simplified scheme of molecular pathways modulated by natural compounds is present in Figure 5.

Withaferin A (WA), isolated from the *Withania somnifera*, an Indian medicinal plant, is among the compounds studied against MPM. This steroidal lactone exerts potential anti-inflammatory, anti-angiogenic, and anti-metastatic properties that target multiple molecular pathways, including NF-κB, PI3K/AKT/mTOR signaling pathways, and the generation of ROS, mitochondrial dysfunction, and proteasome activity inhibition [72]. Several molecular alternations were observed in both in vivo murine allografts and in vitro patient-derived MPM models after WA treatment. WA caused tumor growth inhibition, involving direct apoptosis, the increase in pro-apoptotic proteins (p38 stress-activated caspase-3, elevated Bax, PARP cleavage, stimulated expression of CARP1/CCAR1), and blockage in the chymotryptic activity of proteasome [73].

Given the recent miRNA targeting MPM therapy, it is impressively attractive that phytochemicals seem to be reasonable miRNA regulatory tools. A broad range of natural compounds has been tested for unique miRNA regulatory properties, including curcumin, EGCG, resveratrol, quercetin, and others [74]. So far, only ursolic acid, presented in leaves, fruits, flowers, and berries, has a documented role in epithelial mesothelioma miRNA expression [75]. In the light of altered gene expression patterns observed after the administration of particular polyphenols and documented in other various cancer types’ miRNA modulatory potential, dietary phytochemicals appeared to be a promising additional option for MPM patients.

The discussed above studies confirm the potential anticancer activity of polyphenols and other natural compounds that can directly induce apoptosis of mesothelioma cells and increase MPM cells’ sensitivity to chemotherapy. The summary of the current results of anticancer treatment strategies for malignant pleural mesothelioma, with natural compounds, is presented in Table 2.

Besides the intensively conducted research on the single or combined treatment of particular polyphenolic compounds, several reports demonstrate the antitumor effects of complex mixtures of natural extracts obtained from parts of plants or isolated from secondary post-production wastes.

The first pioneering work on the nutrient mixture composed of green tea extract, vitamin C, amino acids (L-arginine, L-lysine, and proline) and microelements, manganese, and copper was reported by Roomie et al. They revealed a potential therapeutic application of well-balanced supplementation against the MPM cells [76]. The authors proved the inhibitory effect of this composition on the MMPs activity and the level of their secretion by mesothelial cells in a dose-dependent manner (below 500 µg/mL), at the same time ensuring the strengthening of the extracellular matrix components and affecting the ability of malignant mesothelial cells to invade the matrix and expand. Moreover, Roomie’s team highlighted the safety issue of the tested preparation, indicating the lack of side effects on organs and serum enzymes in animal models compared to chemotherapy agents [77]. Likewise, the previously discussed example, Pulito et al., revealed encouraging results in both in vitro and in vivo models of malignant pleural mesothelioma cells treated with artichoke leaf (*Cynara scolymus*) extract [78]. Treatment with the artichoke leaf extract, enriched in phenolic compounds, strongly affected MPM cell growth by inducing apoptosis and impaired the migration and invasion of mesothelioma cells in the first 12 h after administration. Furthermore, by introducing the extract into the diet, these authors observed significantly reduced MPM tumor size in mouse xenografts, this affected the engraftment of the MPM cell line after *C. scolymus* extract treatment. Recently, we reported that a mixture of polyphenols obtained from *Oenothera paradoxa* seeds (evening primrose extract, EPE) revealed anti-invasive effects against MPM cells [79]. Evening primrose (*Oenothera paradoxa* Hudziok), belonging to the *Oenothera* species, is used in Europe for edible oil production. It is well-established that the oil obtained from *Oenothera* species seeds is rich in γ-linolenic acid and exhibits anti-diabetic and anti-inflammatory effects. Nowadays, it is widely used in hypercholesterolemia and obesity treatment and the therapy of atopic eczema, premenstrual syndrome, and multiple sclerosis [80]. Several studies on the determination of valuable compounds of defatted EPE seeds demonstrated significantly high polyphenols content comparable to polyphenols presented in extracts obtained from green tea or grapefruit seeds [81]. Pharmacological studies have revealed that the extracts obtained from *O. biennis* and *O. paradoxa* have biomedical properties, demonstrating the antioxidant and iron (II) chelating activity [82,83]. Analysis of the individual components of *Oenothera paradoxa* seeds extracts led to the identification of biologically active compounds, including gallic acid and PGG, which, apart from anti-inflammatory and antioxidant properties [84,85], displayed a selective pro-apoptotic activity against certain types of tumors [29,86]. The influence of EPE from evening primrose post-industrial seeds on cancer proliferation has been studied before [87,88,89]. Depending on the extraction method, these preparations vary in the total amount of polyphenols and composition; nevertheless, the presence of the main polyphenol compounds, including gallic acid, epicatechin, PGG, and procyanidins, make the extract valuable for therapeutic purposes. So far, research conducted on human melanoma, colon cancer, and Ehrlich ascites cells confirmed that both EPE and its main compound PGG could induce apoptosis in these cells in a pro-oxidative manner [90,91,92]. On the other hand, Lewandowska et al. have shown potential anti-metastatic properties of EPE against prostate and breast cancer types, namely by inhibiting the type IV collagenases (MMP-2 and MMP-9) responsible for cell migration, invasiveness, and metastasis [93]. Our latest study [79] revealed that EPE possesses the anti-metastatic specificity toward invasive MPM cancer cell types (MSTO-H211 and JU77). The extract, containing ellagic acid, gallic acid, (+)-catechin, PGG, and procyanidin B2, arrested invasive cells in the G2/M phase, negatively correlated with the migration of MPM cells, and inhibited the secretion of matrix metalloproteinase-7 (MMP-7), the latter factor directly engaged in the invasion process and metastasis. Considering that MPM is a cancer with one of the worst prognoses, which increases rapidly in the advanced stage, this property of EPE makes it valuable in inhibiting the progression and worsening of this malignancy. It was reported previously by Jaszewska et al. that the *O. paradoxa* extract containing PGG and procyanidins potentiates the cytotoxicity of the chemotherapeutic agent vincristine in human metastatic melanoma cells (HTB-140) [94]. The authors confirmed the extract’s potency in more invasive cells than hepatoma cells (HepG2), indicating the potential pro-oxidative activity of its components on tumor cells that bear elevated intracellular levels of the reduced form glutathione (GSH). Following this premise, we evaluated the activity of individual constituents of EPE preparation. We did not observe the potency of a single use of phenolic compounds (unpublished data), raising the rationale for the extensive use of natural extracts. Our ongoing results provide evidence for further in-depth analysis of the molecular mechanisms of EPE in MPM tumor-growth suppression. In the light of recent reports, it seems that the polyphenol-rich extracts obtained from *Oenothera* sp. have a dual biological effect, acting as an antioxidant and a protective agent toward normal cells and revealing anticancer activity toward MPM cells, with concomitant support of the standard chemotherapy.

Regarding the significant limitations to the successful treatment of malignant pleural mesothelioma, including severe side effects and chemoresistance, phytochemicals seem to be a reasonable approach to alleviating the aggressiveness of standard doses of chemotherapeutic agents while sensitizing cancer cells to treatment and protecting healthy tissues and organs. The involvement of natural extracts in the MPM treatment regimen might improve the tolerance and efficacy of the therapy.

## 5. Conclusions

Malignant pleural mesothelioma therapy is still an emerging issue due to the ineffective treatment options, especially in late-diagnosed advanced-stage patients. The main frontline of the MPM treatment strategy is surgery, curative intents, or palliative chemotherapy. New therapeutic approaches involving targeted strategies and immunotherapies and novel treatment options, considering combinatory treatment and involvement of natural compounds and plants-origin extracts, are still needed. Herein, we discussed the best with today’s knowledge of the research studies that consider targeted therapies and immunotherapies in MPM tumor treatment, focusing on the natural compounds’ phytotherapy. The beneficial effects of polyphenols and plant-derived extracts, including *Oenothera paradoxa* preparation, on the treatment of MPM neoplasm, presented in this work, provide a new opportunity to engage pure nature compositions in inhibiting MPM cancer progress. We take a step further to influence this malignancy with the in-depth coverage of the molecular and cellular pathways involved in polyphenols activity. We hope that, as in the case of other solid tumors, including melanoma and non-small cell lung carcinoma, MPM patients will soon gain benefits from long-lasting disease control and a better prognosis for the future.

## Figures and Tables

**Table 1:** Major classes of polyphenols and their representatives.

**Flavonoids**	**Flavonols**	**Flavones**		**Flavanols**	
	quercetin 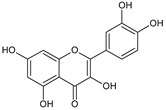	luteolin 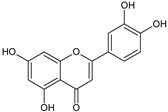		catechin 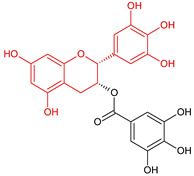 epigallocatechin-3-gallate (EGCG)	
	**Anthocyanidins**	**Isoflavones**		**Flavanones**	
	cyanidin 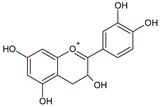	genistein 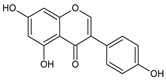		hesperidin 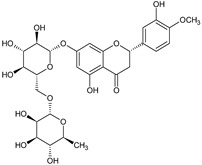	
**Phenolic acids**	gallic acid 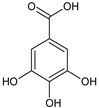	caffeic acid 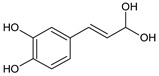		protocatechuic acid 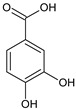	
**Stilbenes**	resveratrol 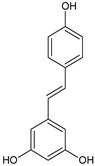	**Lignans**	pinoresinol 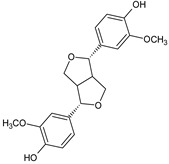	**Other polyphenols**	curcumin 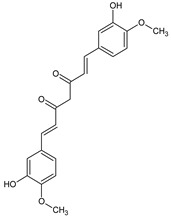

**Table 2:** Studies on polyphenolic compounds in the treatment of malignant pleural mesothelioma.

Compound	Molecular Target Effect	Effect	Reference
Curcumin	↑ Bax proteins and caspase-9 expression; Induction of XAF1, CARP1/CCAR1, SULF1	Stimulation of pro-apoptotic factors; induction of novel apoptosis signaling transducers;	[41] [44]
		reduction of tumorigenic properties of MPM cells in an animal model	[43]
Epigallocatechin-3-gallate (+ascorbate and gemcitabine)	↑ ROS generation	Impairment of mitochondrial membrane potential;	[51]
	↓ NF-κB activation (↓ p65 subunit)	restoring programmed cell death;	[53]
	pro-apoptotic ER stress (UPR shift and GRP78 accumulation in ER)	induction of pro-apoptotic signals	[54]
Resveratrol (+cisplatin or +clofarabine)	↑ROS generation in mitochondria ↓ Sp1 protein level ↓ expression of Sp1 regulatory proteins: p21, p27, cyclin D1, Mcl1, and survivin ↑ caspase-3 activation↓ anti-apoptotic Bcl-xL ↑ pro-apoptotic Bax	Activation of the apoptotic pathway; MSTO-H211 cells sensitization to the antitumor effect of clofarabine and cisplatin	[55,56,57] [60,61]
Quercetin (+ cisplatin)	↓ Sp1 protein and mRNA level ↑ caspase-3 signaling ↓ anti-apoptotic Bcl-xL ↑ pro-apoptotic Bax	S-phase arrest; activation of the apoptotic pathway; sensitization to the antitumor effect of cisplatin	[62,63,64]
Gallic acid	↓ expression of cyclin D and Bcl-2 genes; altered expression of p21	G1 phase arrest; mitochondria dysfunction; apoptosis	[65]
Cafestol 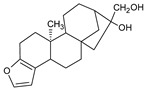 Kahweol 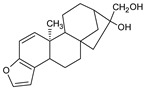	↓ Sp1 protein level ↓ expression of Sp1 regulatory proteins: cyclin D1, Mcl1, and survivin ↑ caspase-3 signaling ↓ anti-apoptotic Bcl-xL ↑ pro-apoptotic Bax, Bid	Growth arrest of MSTO-H211 cellsat Sub-G1 phase; activation of apoptotic signals	[66]
Hesperidin 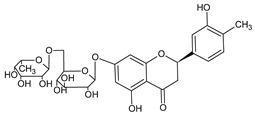	↓ Sp1 mRNA and protein level ↓ expression of Sp1 regulatory proteins: cyclin D1, Mcl1, and survivin ↑ caspase-3 signaling ↓ anti-apoptotic Bcl-xL ↑ pro-apoptotic Bax, Bid	Growth arrest of MSTO-H211 cellsat Sub-G1 phase; activation of apoptotic signals	[68]
Honokiol 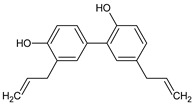	↓ Sp1 mRNA and protein level ↓ expression of Sp1 regulatory proteins: cyclin D1, Mcl1, and survivin ↑ caspase-3 signaling ↓ anti-apoptotic Bcl-xL ↑ pro-apoptotic Bax, Bid	Proliferation inhibition; growth arrest of MSTO-H211 cellsat Sub-G1 phase; activation of apoptotic signals	[69]
Licochalcone A 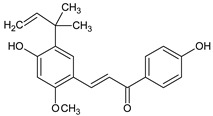	↓ Sp1 mRNA and protein level ↓ expression of Sp1 regulatory proteins: cyclin D1, Mcl1, and survivin ↑ caspase-3 signaling ↓ anti-apoptotic Bcl-xL ↑ pro-apoptotic Bax, Bid	Growth arrest of MSTO-H211 and NCI-H28 cells at Sub-G1 phase; depolarization of mitochondria membrane; activation of apoptotic signals	[71]
Withaferin A 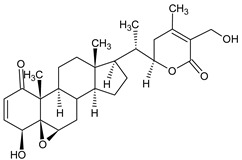	↑ p38 stress-activated caspase-3, ↑ Bax protein, PARP cleavage, induction of CARP1/CCAR1	Increase of pro-apoptotic proteins; blockage in the chymotryptic activity of proteasome	[73]
